# Spontaneous resolution of post-traumatic chronic subdural hematoma: a case report

**DOI:** 10.11604/pamj.2017.28.167.13944

**Published:** 2017-10-20

**Authors:** Hakan Yilmaz, Osman Boyali, Ibrahim Burak Atci, Umit Kocaman

**Affiliations:** 1 Usak University Education and Research Hospital, Department of Neurosugery, Usak, Turkey; 2 Duzce Ataturk State Hospital, Department of Neurosugery, Duzce, Turkey; 3 Istanbul Education and Research Hospital, Department of Neurosugery, Istanbul, Turkey; 4 Izmir Cigli Education and Research Hospital, Department of Neurosugery, Izmir, Turkey

**Keywords:** Antiaggregation therapy, chronic subdural hematoma, spontaneous resolution

## Abstract

Chronic subdural hematomas often occurs in late middle and old age following trivial head trauma. Surgical intervention is the first treatment option in chronic subdural hematomas which compressed the cerebral parenchym. Hematoma may be calcified or ossified in untreated patients. Spontaneous resolution of post-traumatic chronic subdural hematoma is a rare event. Spontaneous resolution is rarer if the subdural hematoma is bilateral. In the literature, this condition is reported mostly in patients with idiopathic thrombocytopenic purpura. Here, we present a case of spontaneously resolved post-traumatic bilateral chronic subdural hematoma within a period of one month in a 55-year-old male and we discuss the probable mechanisms of pathophysiology in the spontaneous resolution of chronic subdural hematoma.

## Introduction

Spontaneous resolution of chronic subdural hematoma (CSDH) is a rarer event, it is very rarer if the subdural hematoma is bilateral and possible mechanisms are still controversial. Hematoma may be calcified or ossified in untreated patients. Foreseeing which hematomas are likely to resolve spontaneously may prevent unnecessary surgery [1,2].

## Patient and observation

**Figure 1:**
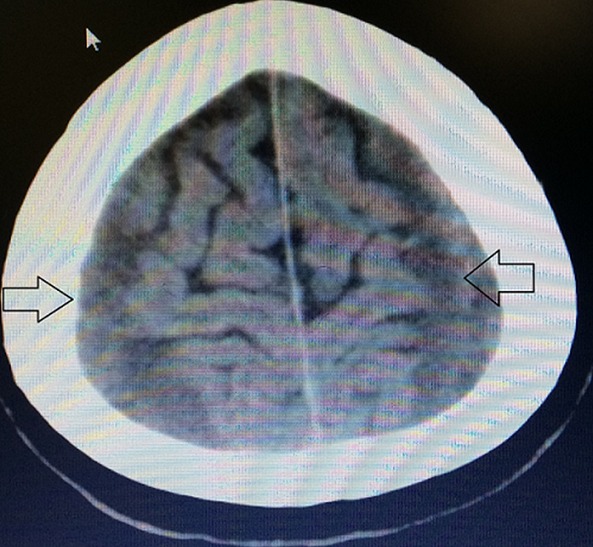
Initial cranial CT scans show a 20 mm thick low-isodensity chronic subdural hematoma at bilateral frontotemporoparietal region

**Figure 2:**
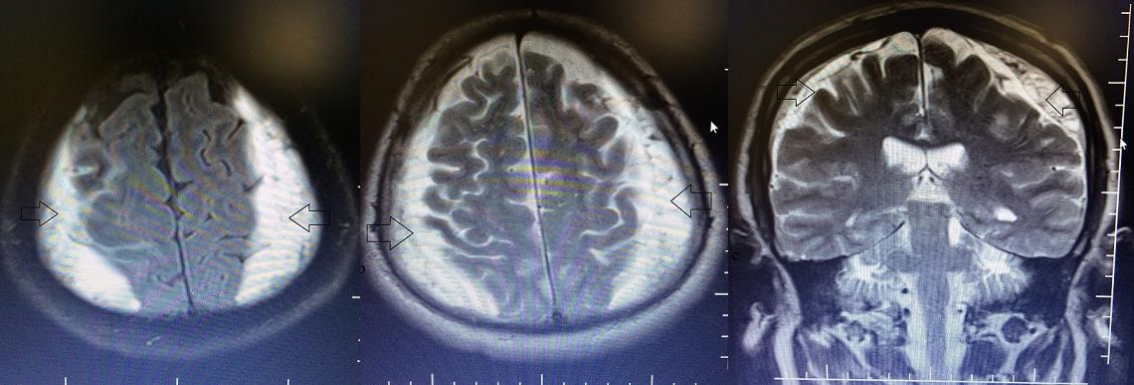
Bilateral frontotemporoparietal hyperintense images on T1 and T2-weighted axial and coronal MRI scans at initial presentation

**Figure 3:**
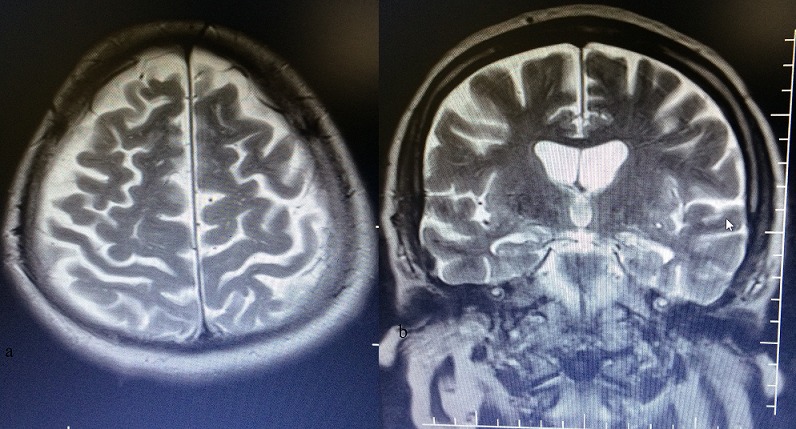
Follow-up MRI scans 1 month later showed spontaneous resolution of bilateral subdural hematoma

A 55 year old man, who suffered a doubtful head trauma one month ago, was admitted to our hospital with headache and debility complaints. We did not find any pathology on his neurologic examination. In his past medical history, he had undergone coronary bypass surgery and used regularly antiaggregation therapy (acetylsalicylic acid 100 mg/day) for 2 years. We performed cranial computed tomography (CT) that revealed hypo-isodense collections of 2 cm thickness at bilateral frontotemporoparietal region (Figure 1) suggestive of bilateral CSDH. Cranial magnetic resonance imaging (MRI) at T1 and T2 axial and coronal sequence revealed bilateral frontotemporoparietal hyperintense lesion (Figure 2) defined as CSDH. There was no midline shift and compression of the ventricules. Emergency surgery was recommended to the patient due to bilateral hematoma which was larger than 1cm. After cardiology consultation, antiaggregation theraphy was stoped and enoxaparin sodium 20 mg/day was started. However, the patient and his relatives were not willing to undergo surgery and he was discharged with anti-edema treatment and antiepileptic drugs. One month later, he had visited to the cardiology policlinic and he had referred to our policlinic for control examination. The patient had no complaints and neurological examination revealed no pathological findings. Control cranial MRI revealed that almost all of bilateral CSDH had resolved spontaneously (Figure 3). He was symptomatically treated and discharged with suggestions.

## Discussion

Surgical treatment of subdural hematoma is necessary in the presence of neurologic deficit, significant mass effect and mid-line shift due to the hematoma. Surgical results were generally excellent [1]. In the literature, spontaneous resolution of CSDH has rarely been reported. It is very rarer if the subdural hematoma is bilateral [2]. Foreseeing which hematomas are likely to resolve spontaneously may prevent unnecessary surgery. Radiologic properties of spontaneously resolved CSDH are small size, minimal mass effect, settlement in the frontal region, low-iso density of hematoma and absence of mid-line shifting. Moreover, low density line indicating CSF between the hematoma and cortex is a finding that supports spontaneous resolution. Clinically, spontaneous resolution tends to occur more frequently in asymptomatic patients or patients having mild neurological symptoms. Spontaneous resolution of a CSDH, which presented with progressive neurologic symptom and revealed significant mass effect, has rarely been reported in the literature [3]. In our case, hematoma was iso-hypodense on CT, hematoma has minimal mass effect and the patients has mild neurological symptoms. In addition, there was a low density line indicating CSF between the hematoma and cortex on the CT.

Various theories have been suggested to explain the mechanisms of resolution of CSDH. In 1981, Kawano and Suzuki suggested that smooth muscle cells in the outer membrane might produce collagen that supports the membran, reducing the fragility of the membrane, thus they thought smooth muscle cells might play a role in the resolution of CSDH [3,4]. In the advancing years, Yamashima et al. proposed that the platelet formation in the microcapillaries may cause this spontaneous resolution by reducing microhemorrhages [5]. Nakamura et al. claimed that the decreased fibrinolytic activity of the hematoma and its capsule might have caused spontaneous resolution [4,6]. Nomura et al. showed that decreased fibrinolytic activity and decreased predisposition to rebleeding have detected in the patients with low density CSDH on the CT scan [7]. Giuffrè claimed that hormonal, mechanical, hematogenic and vasogenic factors have an impact on the pathogenesis of these hematomas [8]. Glover and Labadie suggested that corticosteroids inhibit the formation of membrane and by this way decrease the size of hematoma in an animal model [9]. Finally, Lee maintaned that maturation of the neomembrane and stabilisation of neovascularation might affect the spontaneous resolution [10].

## Conclusion

Recovery mechanisms of CSDH in patients with operated or nonoperated are nearly the same. More importantly, predicting which hematomas tend to resolve spontaneously may prevent unnecessary surgery. In the literature, spontaneous resolution tends to occur if the patient is asymptomatic and if the hematoma has small size, low or isodensity, CSF line between hematoma and cortex and minimal mass effect without mid-line shifting.

## Competing interests

The authors declare no competing interests.
